# Prison Reform

**Published:** 1898-04-23

**Authors:** 


### Modern Sociology.

### PRISON REFORM.

### What the Authorities Say.

Within the last few weeks certain sensational statements have been made in the House of Commons as to the treatment of prisoners within this realm, which have naturally caused considerable dismay and anxiety on the part of many philanthropic persons who take an interest in the struggling members of that lower stratum of society, whence criminals are mainly drawn. The effect of one somewhat imaginative speech has been to overwhelm the governors of our convict prisons with visits from members of Parliament and others anxious to judge for themselves, and who find to their surprise that the charges of cruelty and unwarrantable harshness of discipline, vanish into thin air when confronted in the scenes where they are supposed to exist. That some improvements in our prison administration may be advantageously carried out is well recognised; but that is no reason why unfair and even false statements should be left to harass and vex the minds of those who have not the opportunity of forming a personal judgment. We must premise that some of the descriptions of ill-treatment of prisoners given in the House of Commons seemed to apply to Ireland alone, and with the condition of gaols in that uneasy island we do not profess to have an actual personal acquaintance, although we believe that the wise and humane desire to deal justly by criminals which prevails in England is most fully shared by those in authority in the sister country. It is, however, of English prisons that we have to speak, and in which our readers are likely to take the greatest interest.

Now, one of the principal charges against them, on which the orator in the House enlarged, was the insufficiency and bad quality of the food provided for the prisoners. This charge against the quality of the food is certainly false so far as English prisons are concerned. The food may not be very palatable, especially to those who have been accustomed to indulge themselves in the most savoury morsels and luxurious feeding they could obtain, but it is perfectly good and wholesome; in fact, the bread, piled up in mountains of loaves at Wormwood Scrubs, is of an unusually excellent type, which could not be complained of in any gentleman's house. Be it remembered, also, that any prisoner who considers he has reason to object to the quality of his breakfast or dinner has a perfect right to make a complaint on the subject, which the officer in charge instantly reports to the governor, and the matter is carefully inquired into without delay. If it is found that the prison cook has been in fault he is censured, and the error rectified at once; but, as a rule, any such charge that has been inquired into has proved to be utterly groundless, and has generally been the result of a captious temper or a malicious desire to give trouble to the authorities. Any idea that the food is not of a perfectly good and proper description may be dismissed, and most frequently disproved by the fact that the great majority of the prisoners have the appearance of being infinitely better in health and more robust when they leave the gaol than when they entered it. With regard to the charge of the insufficiency of the food—there is more ground for the asseveration—but there are certain facts connected with it which not only lessen the gravity of the imputation, but to a great extent justify the course taken by the authorities. First, as to the reality of the charge—the Parliamentary speaker entirely ignored the fact that the medical officer in every prison is absolutely omnipotent as to diet, he can order what he pleases to the extent of luxuries, both in quantity and quality, which could hardly be exceeded even for the most prosperous invalids at large in their own abodes; and he can do this not only for prisoners ill enough to be in the infirmary, but for any man or woman going through the ordinary prison work who seems to him below par, and, from constitutional delicacy or other reasons, requiring to be built up into better health. No one can even remonstrate with him on his right to do this to any extent he pleases, and to continue it till the patient's sentence actually expires; and considering that he is bound to visit every prisoner daily, it is not likely that any serious effect from a restricted diet will ever be allowed to continue without his aid.

As regards perfectly strong and healthy prisoners, it is true that they could probably, most of them, consume more than the amount of food provided for them. But a prison is supposed to be a place of temporary punishment for breaking the laws of the land, and often for outrages against the lives and the property of law-abiding citizens. Would it not be a very great injustice on honest, hard-working men who maintain their integrity, even when, from adverse circumstances, their families may be almost starving, if the criminals in the gaols were to be in the enjoyment of a bountiful and unfailing diet, with long fixed hours of rest in a clean bed, and assiduous attention given to their wants in every way? Most of that they do enjoy now, with the single exception that they do not have quite so much to eat as their very large appetites could appropriate. Is it right to make a prison infinitely more comfortable and well supplied than the poor cottage of the honest workman? Already we often hear in our police-courts that tramps would rather go to the prison than to the workhouse. If our orator's views were to be carried out, the gaol would soon become preferable to the scantily provided home of the guiltless.

One more of the speaker's charges we must briefly notice. He fiercely reprobates the practice of firing at convicts escaping from Dartmoor or Portland, and their attempts, when captured, being visited with corporal punishment. He forgets that their first effort for liberty often consists in murdering their warders or injuring them for life, and that if no serious penalty followed such proceedings the prison service would become a profession of the utmost danger to the worthy, high-principled men who at present engage in it.

### A LEGAL DIFFICULTY.

An interesting and curious case has just been decided in the Chancery Court, arising out of some property to be used as the site of the Heysham Convalescent Home about to be built for the benefit of the people of Bradford. Apparently after the contract was made the defendant in the action came to the conclusion that he had parted with his property at too cheap a rate. Mr. Justice Stirling decided otherwise, and the property has had to be handed over to the authorities at the original price agreed upon, £8,000.

